# Association of multiple rib fractures with the frequency of pneumonia in the post-resuscitation period

**DOI:** 10.1016/j.resplu.2022.100267

**Published:** 2022-07-01

**Authors:** Yasuyuki Kawai, Keisuke Takano, Keita Miyazaki, Koji Yamamoto, Yusuke Tada, Hideki Asai, Naoki Maegawa, Yasuyuki Urisono, Keigo Saeki, Hidetada Fukushima

**Affiliations:** a Department of Emergency and Critical Care Medicine, Nara Medical University, Nara, Japan; b Department of Epidemiology, Nara Medical University School of Medicine, Nara, Japan

**Keywords:** Out-of-hospital cardiac arrest, Cardiopulmonary resuscitation, Pneumonia, Thoracic injury, Rib fractures, CA, cardiac arrest, CPR, cardiopulmonary resuscitation, CT, computed tomography, HR, hazard ratio, OHCA, out-of-hospital cardiac arrest, ROSC, return of spontaneous circulation, SSRF, surgical stabilisation of rib fractures, TH, therapeutic hypothermia

## Abstract

**Purpose:**

Successful cardiopulmonary resuscitation is associated with a high incidence of chest wall injuries. However, few studies have examined chest wall injury as a risk factor for respiratory complications after cardiopulmonary resuscitation. Therefore, herein, we investigated the association of multiple rib fractures on the incidence of post-resuscitation pneumonia.

**Methods:**

This single-centre retrospective cohort study enrolled adult, nontraumatic, out-of-hospital cardiac arrest patients who maintained circulation for more than 48 h between June 2015 and May 2019. Rib fractures were evaluated by computed tomography on the day of hospital admission. The association with newly developed pneumonia within 7 days of hospitalisation was analysed using a Fine-Gray proportional hazards regression model adjusted for the propensity score of multiple rib fractures estimated from age, sex, presence of witnessed status, bystander CPR, initial rhythm, and total CPR time and for previously reported risk factors for pneumonia (therapeutic hypothermia and prophylactic antibiotics).

**Results:**

Overall, 683 patients with out-of-hospital cardiac arrest were treated; 87 eligible cases were enrolled for analysis. Thirty-two (36.8%) patients had multiple rib fractures identified on computed tomography, and 35 (40.2%) patients developed pneumonia. The presence of multiple rib fractures was significantly associated with a higher incidence of pneumonia, consistently both with and without adjustment for background factors (unadjusted hazard ratio 4.63, 95% confidence interval: 2.35–9.13, *p* < 0.001; adjusted hazard ratio 4.03, 95% confidence interval: 2.08–7.82, *p* < 0.001).

**Conclusions:**

Multiple rib fractures are independently associated with the development of pneumonia after successful resuscitation.

## Introduction

Cardiac arrest (CA) is the leading cause of death in many countries. Successful resuscitation is dependent on immediate chest compressions.1., 2. Cardiopulmonary resuscitation (CPR) that produces adequate coronary perfusion pressure from hard and fast chest compressions is associated with better neurological outcomes.3., 4. However, several authors have indicated that chest compressions can cause various complications.5., 6. Rib fractures are the most common complications, and several studies have reported an increase in rib fractures as harder and faster chest compressions are recommended.6., 7., 8.

During the post-resuscitation period, a patient is exposed to post-CA syndrome, a systemic inflammatory condition, and is highly susceptible to infections.9., 10., 11. Pneumonia is the most frequent complication of chest compression, with a reported prevalence of 29–70%.9., 10., 11., 12., 13. Although pneumonia has not been evaluated as a risk factor for poor prognosis after resuscitation. It is significant because it prolongs the duration of ventilation use and hospital stay and increases health care costs.12., 13., 14., 15.

Multiple rib fractures caused by blunt trauma due to events such as road traffic accidents increase the risk of developing pneumonia, and it worsens the prognosis.^16^ Therefore, analgesic interventions are applied, and surgical stabilisation of rib fractures (SSRF) is considered.17., 18. Despite these concerns, rib fractures caused by chest compressions have not received adequate attention in research as a risk factor for the development of pneumonia after resuscitation. In particular, the impact of multiple rib fractures on the development of pneumonia in post-resuscitation patients has not been studied; therefore, there is insufficient information regarding analgesia and SSRF after resuscitation.

We hypothesised that the occurrence of multiple rib fractures after resuscitation would be a risk factor for the development of pneumonia during the post-resuscitation period. If multiple rib fractures increase the incidence of pneumonia in the post-resuscitation period, it will provide the same rationale for the need for therapeutic intervention as that considered for multiple rib fractures due to blunt trauma.

## Methods

### Study design

This single-centre, retrospective observational study was approved by the institutional ethics committee of Nara Medical University (No. 2328). The need for informed consent was waived owing to the retrospective observational nature of the study. Because this was an exploratory study, we could not establish a required sample size.

### Data collection

We investigated patients with out-of-hospital cardiac arrest (OHCA) treated at our institution between June 2015 and May 2019. Data were obtained from electronic medical records recorded in the Utstein style.^19^ The inclusion criteria were age ≥18 years, diagnosis of nontraumatic CA, and subsequent achievement of sustained spontaneous circulation or extracorporeal circulation for >48 h. The exclusion criteria were confirmed pre-existing pneumonia by chest computed tomography (CT) on arrival at the hospital, no chest CT evaluation after hospital admission, and missing data regarding time events for CPR. We collected data on the characteristics and potential risk factors associated with pneumonia after CPR, including age, sex, witnessed status, bystander CPR, initial rhythm, total CPR time (including bystander CPR), use of therapeutic hypothermia (TH), use of muscle relaxants, use of prophylactic antibiotics within 24 h of CA, use of mechanical ventilation, and number of rib fractures.

### Diagnosis

Rib fracture was diagnosed using chest CT performed before admission to the intensive care unit. All CT images were acquired with a 64-row helical CT system (Optima CT660; GE Healthcare, Waukesla, WI, USA). The scan variables were as follows: 120 kVp; auto mA; rotation time, 0.5 s; helical pitch, 0.531; and image noise, SD10. Coronal, sagittal, and three-dimensional reconstructed images were obtained and used for the diagnosis. The frequency of the occurrence of rib fractures after resuscitation varies widely among different reports.^20^ The diverse injury morphology of rib fractures induces diagnostic variability among examiners. Therefore, in this study, all the types of fractures, displaced, non-displaced, incompletely displaced, and buckle fractures,^20^ were included. To ensure consistent rib diagnostic criteria were used in this study, the diagnosis was made by emergency physicians who understood the diagnostic criteria and were educated in surgical findings. Previous studies have reported that most rib fractures that occurred after resuscitation were bilateral and multiple20., 21.; thus, we defined three or more rib fractures as multiple rib fractures. We also defined a flail chest as three or more consecutive rib fractures in two or more locations, forming a flail segment.^16^

We evaluated the development of pneumonia during the first 7 days of hospitalisation.^13^ In general, pneumonia after resuscitation is difficult to diagnose accurately on a chest X-ray due to aspiration or lung parenchymal injury resulting from the resuscitation.^15^ Therefore, we excluded patients with aspiration and lung parenchymal injury on chest CT scans obtained at admission. Moreover, we made a diagnosis of pneumonia only in patients who fulfilled all commonly used diagnostic criteria: clinically relevant findings at auscultation, decreased oxygenation that could not be explained by pulmonary oedema or atelectasis, new consolidation on a chest radiograph persistent for ≥48 h after admission, and an increase in airway secretions.^22^ There was no microbiological diagnosis in all cases, but samples collected within 7 days of admission were used as a reference for diagnosis.

Rib fractures and pneumonia were diagnosed by two independent emergency physicians with more than 8 years of experience but who were blinded to the diagnoses of pneumonia. Any diagnostic disagreements were resolved by consensus between the physicians.

### Post-resuscitation care

According to our standardised protocol, patients were managed with sedation, analgesia, and mechanical ventilatory support, adhering to resuscitation guidelines.^23^ TH was indicated for comatose survivors. The exclusion criterion for TH was shock despite vasopressor use (systolic blood pressure <90 mmHg). A central venous catheter and a gastric tube were inserted for all patients who underwent TH. The core body temperature was maintained at 33 °C for 24 h; subsequent rewarming was at a rate of 0.25–0.5 °C per hour and maintained at 37 °C for another 24 h with an Arctic Sun® Temperature Management System (Bard, BD, Covington, GA, USA).

### Statistical analysis

Categorical and continuous variables are expressed as *n* (%) and median (interquartile range), respectively. Continuous and categorical data are reported as the median (interquartile range) and frequency (percentage), respectively. For univariate comparisons, Mann–Whitney U tests were used for nonparametric continuous variables, whereas Fisher’s exact tests were used for categorical variables. The diagnostic agreement rate for multiple rib fractures using CT examination was evaluated using the kappa value.

To calculate the propensity score for multiple rib fractures, we constructed a logistic regression model that includes background factors such as age, sex, presence of witnessed status, bystander CPR, initial rhythm (ventricular fibrillation or pulseless ventricular tachycardia) and total CPR time^24^ as independent variables.

To estimate the hazard ratio by considering competing risk of death from other than pneumonia, we constructed the Fine-Gray proportional hazards regression model. The model was adjusted for previously reported risk factors for pneumonia (therapeutic hypothermia and prophylactic antibiotics) and propensity score for multiple rib fractures.

Based on previous studies about risk factors for pneumonia, we listed four variables as confounding factors: mechanical ventilation,11., 12., 13. TH,^25^ use of muscle relaxants,^26^ and use of prophylactic antibiotics.13., 27. However, mechanical ventilation could not be adjusted as most patients (90.8%) were using it because TH and use of muscle relaxant correlate with each other and are at risk of multicollinearity; TH was selected as the variable in this study based on the discussion in several previous studies.9., 14., 22., 25. The variables finally selected for adjustment were the presence of multiple rib fractures, TH, and use of prophylactic antibiotics. The hazard ratios of multiple rib fractures for the development of pneumonia were obtained by adjusting for these factors, with and without adjustment by the propensity score for multiple rib fractures. The same analysis was performed as a sensitivity analysis by replacing TH with muscle relaxants as a variable.

A *p*-value of <0.05 denoted a statistically significant difference. R version 4.0.2 (R Foundation for Statistical Computing, Vienna, Austria) was used for the statistical analysis.

## Results

**Fig. 1:**
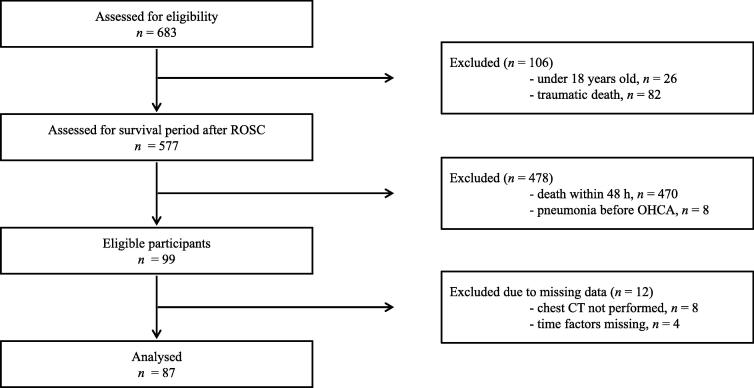
**Flow diagram of participants’ inclusion** CT, computed tomography; OHCA, out-of-hospital cardiac arrest; ROSC, return of spontaneous circulation.

During the study period, 683 patients with OHCA underwent resuscitation and were transferred to our hospital. Initially, we excluded patients aged <18 years and with CA following trauma. Next, patients who died within 48 h or who had confirmed pre-existing pneumonia based on chest CT findings at hospital arrival were excluded. We further excluded 12 patients with missing time data or who did not undergo chest CT. Finally, 87 patients were included in the study analysis (Fig. 1). Patient background and the comparison of the presence of multiple rib fractures are shown in Table 1. The median age of the overall group was 67 years; men accounted for 63.2% of all patients. Multiple rib fractures were found in 32 patients (36.8%), and 35 patients (40.2%) developed pneumonia within 7 days after hospitalisation. The diagnostic agreement rate for multiple rib fractures was kappa: 0.81 (95% confidence interval [CI]: 0.68–0.93). Compared to patients in the non-multiple rib fracture group, those in the multiple rib fracture groups were older, required mechanical ventilation more often, had more indications for TH, and required muscle relaxants more often.

**Table 1:** Patient background and propensity score-adjusted factors.

	All participants			
		Multiple rib fractures		
Variables	Overall	Yes	No	*p* value
	*n* = 87	*n* = 32	*n* = 55	
Age, years	67.0 [58.5, 75.0]	70.5 [62.8, 77.0]	66.0 [56.0, 74.0]	0.04
Male sex, *n* (%)	55 (63.2)	18 (56.2)	37 (67.3)	0.36
Cardiac cause of cardiac arrest, *n* (%)	55 (63.2)	19 (59.4)	36 (65.5)	0.65
Body mass index	21.6 [18.0, 24.6]	20.7 [17.6, 25.3]	22.2 [18.9, 24.5]	0.41
Witness, *n* (%)	65 (74.7)	25 (78.1)	40 (72.7)	0.62
Bystander CPR, *n* (%)	47 (54.0)	18 (56.2)	29 (52.7)	0.83
VF or pulseless VT as initial ECG rhythm, *n* (%)	31 (35.6)	10 (31.2)	21 (38.2)	0.64
Received shock, *n* (%)	35 (40.2)	13 (40.6)	22 (40.0)	1
Duration of CPR, minute	26 [15, 39]	34 [22, 43]	21 [13, 35]	0.05
Side with the ribs fractured, *n* (%)				
Bilateral	29 (33.3)	29 (90.6)	0 (0.0)	<0.001
Right	9 (10.3)	2 (6.2)	7 (12.7)	
Left	4 (4.6)	1 (3.1)	3 (5.5)	
Sternal fracture, *n* (%)	4 (4.6)	4 (12.5)	0 (0.0)	0.02
Number of right side rib fractures	0 [0, 3]	4 [3, 5]	0 [0, 0]	<0.001
Number of left side rib fractures	0 [0, 3]	3 [3, 4]	0 [0, 0]	<0.001
Multiple rib fractures, *n* (%)	32 (36.8)	32 (100.0)	0 (0.0)	
Flail chest, *n* (%)	23 (26.4)	23 (71.9)	0 (0.0)	<0.001
Mechanical ventilation, *n* (%)	79 (90.8)	32 (100.0)	47 (85.5)	0.02
Length of mechanical ventilation, day	7 [4, 11]	9 [5, 16]	6 [3, 10]	0.03
Extracorporeal circulation, *n* (%)	14 (16.1)	5 (15.6)	9 (16.4)	1
Therapeutic hypothermia, *n* (%)	43 (49.4)	21 (65.6)	22 (40.0)	0.03
Muscle relaxants, *n* (%)	28 (32.2)	15 (46.9)	13 (23.6)	0.03
Prophylactic antibiotics, *n* (%)	45 (51.7)	17 (53.1)	28 (50.9)	1
Sputum culture	37/47 (79)	18/21 (86)	19/26 (73)	0.03
Length of stay in intensive care unit, day	8 [4, 12]	9 [6, 13]	6 [4, 11]	0.046
Length of hospital stay, day	23 [8, 43]	31 [14, 47]	18 [8, 40]	0.16
Observation period, day	24 [8, 44]	31 [13, 48]	19 [8, 39]	0.17
CPC 1/2, *n* (%)	23 (26.4)	3 (9.4)	20 (36.4)	0.01
Development of pneumonia, *n* (%)	35 (40.2)	23 (71.9)	12 (21.8)	<0.001
Mortality, *n* (%)	33 (37.9)	14 (43.8)	19 (34.5)	0.49

Bracketed numbers are ranges. CPR, cardiopulmonary resuscitation; VF, ventricular fibrillation; VT, ventricular tachycardia; ECG, electrocardiographic; CPC, cerebral performance category.

Unadjusted HR of multiple rib fractures for development of pneumonia was significant (HR: 4.63; 95% CI: 2.35–9.13; *p* < 0.001). After adjusting for propensity score, the HR of multiple rib fractures remained significant (adjusted HR: 5.00; 95% CI: 2.48–10.10; *p* < 0.001). Furthermore, the HR of multiple rib fractures was significant even after adjustment for TH and prophylactic antibiotic use, which have been associated with the development of pneumonia in previous studies (adjusted HR: 4.03; 95% CI: 2.08–7.82; *p* < 0.001). (Table 2).

**Table 2:** Hazard ratio for the development of pneumonia in patients with multiple rib fractures using the Fine-Gray proportional hazards regression model.

	Unadjusted model			Adjusted model 1*			Adjusted model 2**		
	HR	95% CI	*p*-value	HR	95% CI	*p*-value	HR	95% CI	*p*-value
Multiple rib fracture	4.63	2.35–9.13	<0.001	5.00	2.48–10.10	<0.001	4.03	2.08–7.82	<0.001
Propensity score	–	–	–	0.46	0.05–4.14	0.48	0.6	0.06–6.30	0.67
Therapeutic hypothermia	–	–	–	–	–	–	3.73	1.66–8.40	0.002
Prophylactic antibiotics use	–	–	–	–	–	–	0.45	0.23–0.87	0.02

* Adusted for propensity score.

** Adjusted for propensity score, therapeutic hypothermia, and use of prophylactic antibiotics. HR, hazard ratio; CI, confidence interval.

A sensitivity analysis adjusted for muscle relaxants instead of TH was performed, and the hazard ratios for multiple rib fractures were similar to those presented in Table 2 (HR: 3.83; 95% CI: 1.94–7.57; *p* < 0.001, adjusted HR: 3.71; 95% CI: 1.83–7.49; *p* < 0.001).

## Discussion

In our study, the frequency of multiple rib fractures was as high as 36.8%, and 91% of the patients had bilateral fractures. To the best of our knowledge, we found, for the first time, that multiple rib fractures were significantly associated with a higher risk of pneumonia independent of propensity score and other confounders (TH and use of prophylactic antibiotics).

Due to dedicated resuscitation efforts to improve the survival outcome of patients with OHCA, a significant number of resuscitated patients with OHCA may sustain multiple rib fracture injuries. Rib fracture is considered a risk factor for pneumonia.28., 29., 30. Several studies have reported other risk factors for the development of pneumonia, such as mechanical ventilation, TH, use of muscle relaxants, and lack of prophylactic antibiotics9., 10., 11., 12., 13., 14., 15., 25., 26., 27. However, no studies have evaluated rib fractures as a risk factor for pneumonia in post-CPR patients. Most risk factors were observed in the multiple rib fracture group than in the non-multiple rib fracture group, which may be related to the development of pneumonia. However, after adjusting for other confounding factors and analysing the results, multiple rib fractures were found to be an independent risk factor for the development of post-resuscitation pneumonia.

The mechanisms through which rib fractures, which occurred during resuscitation after blunt trauma and not after cardiopulmonary arrest resuscitation, cause pneumonia are generally explained by pain,^28^ decreased vital capacity,^31^ and changes in chest wall dynamics that distort the movement of the chest wall muscle.^32^ The frequency of pneumonia occurrence increases with an increasing number of rib fractures, particularly in elderly patients^17^ and patients with flail chest injuries.^18^ Most of the rib fractures observed in this study were bilateral and multiple, and these patients were likely to develop pneumonia within 7 days after hospitalisation. Therefore, the results of this study support the need for careful evaluation of severe chest wall injuries, such as multiple rib fractures, in resuscitated patients with CA to optimise post-resuscitation care.

Interventions for rib fractures in the post-resuscitation period are rarely discussed, and their effectiveness is unknown. However, it is almost impossible to eliminate CPR-related chest wall injuries without decreasing the survival rate of patients with sudden OHCA. Accordingly, physicians should consider this CPR-related complication in the management of post-resuscitation patients with OHCA, and efforts should be made to optimise post-resuscitation care. Early surgical fixation of multiple rib fractures or flail chest due to chest trauma has been reported to improve survival by shortening the duration of mechanical ventilation.17., 18., 33. Further investigation of the increased incidence of pneumonia due to multiple rib fractures, as suggested by this study, would be useful for accurately assessing the previously reported risk factors and considering therapeutic interventions for multiple rib fractures after resuscitation.

Our study had several limitations. First, to minimise the heterogeneity of the assessment factors in this study, we set definitions for diagnosing rib fractures and pneumonia. Rib fractures without dislocation are not always diagnosed. Moreover, the incidence of pneumonia after resuscitation has a large inter-institutional variation, which makes comparison difficult. Therefore, the diagnosis of rib fractures in this study was made by emergency physicians who routinely diagnosed and surgically treated rib fractures after being educated in diagnosis and were blinded to medical background information. Pneumonia was also limited to those that fulfilled all the criteria. However, the presence of rib fractures could not be blinded in the diagnosis of pneumonia based on chest radiographs. Furthermore, the study was limited to the period when the 2015 resuscitation guidelines were applied to make the resuscitation background of the patients as homogeneous as possible. The limitations of this study make generalisation difficult. Therefore, the results of this study will be strengthened by multicentre validation studies using similar criteria.

Second, due to the small number of study participants, we could not adjust for many confounders using multivariable analysis. In this study, we adjusted for previously reported risk factors for the development of pneumonia after cardiac arrest. However, as most cases used mechanical ventilation, mechanical ventilation could not be adjusted for. Therefore, these findings were obtained by adjusting for a few risk factor variables and the propensity score for multiple rib fractures. TH and prophylactic antimicrobial administration were possible partial mediators as they are implemented after exposure to multiple rib fractures. However, these factors have been associated with the development of pneumonia in previous studies; consequently, we considered adjustment for them to be appropriate in this study, which may have underestimated the results of this study. Moreover, although we adjusted for confounding factors as much as possible between multiple rib fractures and the development of pneumonia, we could not completely exclude the possibility that unknown confounding factors may be present.

Finally, post-resuscitation pneumonia in this study was limited to survivors. Previous studies have reported that the frequency of pneumonia in survivors and non-survivors is comparable.12., 25., 27. However, early death patients may not survive until the onset and diagnosis of pneumonia.22., 34., 35. In this study, we evaluated the relationship between multiple rib fractures that warrant therapeutic intervention and the development of pneumonia after resuscitation; therefore, we only examined pneumonia in surviving patients.

## Conclusion

This study showed that rib fractures caused by successful resuscitation increased the incidence of pneumonia after hospitalisation. Considering the development of pneumonia in patients with OHCA who have chest wall injury may optimise post-resuscitation care.

## CRediT authorship contribution statement

**Yasuyuki Kawai:** Data curation, Formal analysis, Investigation, Methodology, Project administration, Validation, Writing – original draft, Writing – review & editing. **Keisuke Takano:** Data curation, Investigation, Writing – review & editing. **Keita Miyazaki:** Data curation, Investigation, Writing – review & editing. **Koji Yamamoto:** Data curation, Investigation, Writing – review & editing. **Yusuke Tada:** Data curation, Investigation, Writing – review & editing. **Hideki Asai:** Data curation, Investigation, Writing – review & editing. **Naoki Maegawa:** Validation, Writing – review & editing. **Yasuyuki Urisono:** Validation, Writing – review & editing. **Keigo Saeki:** Formal analysis, Methodology, Writing – review & editing. **Hidetada Fukushima:** Supervision, Writing – review & editing.

## Declaration of Competing Interest

The authors declare that they have no known competing financial interests or personal relationships that could have appeared to influence the work reported in this paper.
